# MARKETING ISN’T MAGIC: HOW ALTER LEVERAGED THESE BEST PRACTICES TO INCREASE RECRUITMENT AND RETENTION

**DOI:** 10.1093/geroni/igae098.3453

**Published:** 2024-12-31

**Authors:** Fayron Epps, Andy Suggs

**Affiliations:** The University of Texas Health San Antonio, San Antonio, Texas, United States; Reckon Branding, Norcross, Georgia, United States

## Abstract

Dementia-Friendly Faith Villages was launched in 2018 to address the lack of resources and awareness of dementia in African American and faith communities in the State of Georgia. The program struggled to meet recruiting, retention, and program goals in its inaugural year. In response to less-than-desirable progress, the principal investigator partnered with Reckon Branding to define a messaging focus for the program that better connected with each of its key audiences: Church leaders, caregivers, and partner programs within their space. Considering 71% of customers recommend a brand based on their emotional connection to it, Reckon knew they had to tell a story that extended beyond facts and figures. The program was provided with a new verbal and visual focus through internal ideation sessions, audits of competitive programs, and external interviews of Church leadership and caregivers of those with cognitive impairments. Armed with the more memorable name ALTER, a new logo, and more consumer-friendly marketing materials, the team was able to secure 83 partnerships over the next 4 years. This strategic pivot has helped ALTER exceed its partner goals by over 300% and empowered the program to develop consistently impactful marketing materials, which provided improved recruitment reach, increasing the participants from 7,500 to more than 100,000. Thanks to this success, ALTER has extended its reach from Georgia, to many more African American communities of faith across 14 states.

